# Dataset on potential environmental impacts of water deprivation and land use for food consumption in France and Tunisia

**DOI:** 10.1016/j.dib.2019.104661

**Published:** 2019-10-28

**Authors:** Carole Sinfort, Marlene Perignon, Sophie Drogué, Marie Josèphe Amiot

**Affiliations:** a ITAP, Montpellier SupAgro, Irstea, Univ Montpellier, Montpellier, France; b MOISA, Univ Montpellier, CIHEAM-IAMM, CIRAD, INRA, Montpellier SupAgro, Montpellier, France

**Keywords:** INCA2, Tahina, Water stress index, LANCA method, UN comtrade, FAOSTAT

## Abstract

The dataset concerns the environmental impacts of water consumption and land use caused by 1 kg of food item supplied to two Mediterranean countries: France and Tunisia. The dataset takes into account the food items available in France and Tunisia (identified in two national dietary surveys) and their origin. Water consumption and land use surfaces were extracted from existing databases and from national data for animal feed description. Up-to-date available evaluation methods were used to assess the associated impacts. The origin of ingredients was considered to compute impacts on all countries of the world. These data were used in Perignon et al., 2019 [1].

**Table:** Specifications Table

Subject	Environmental Science (General)
Specific subject area	Environmental indicators of food items. Focusses on land use and water consumption.
Type of data	Table
How data were acquired	Data base compilation
Data format	Raw
Parameters for data collection	Data concerns food items consumed in France and Tunisia between 2005 and 2009. Production data and trade statistics are from 2011. Characterization factors used for impacts computation are from 2009 to 2010 for water impacts, 2016 for land use impacts.
Description of data collection	The dataset is an Excel file with two main worksheets: i Data per kg of item and per country of origin for France ii Data per kg of item and per country of origin for Tunisia
Data source location	Food item consumed in France and Tunisia were extracted from national enquiries. Data are given for impacts in every country in the world.
Data accessibility	Repository name: INRA repository https://data.inra.fr/ Data identification number: 10.15454/F37SLV Direct URL to data: https://doi.org/10.15454/F37SLV Licenses of use: <img src = "https://www.etalab.gouv.fr/wp-content/uploads/2011/10/licence-ouverte-open-licence.gif” alt = "Licence Ouverte” height = "100"><a href = "https://Ouverte/Open Licence Version 2.0</a> compatible CC BY
Related research article	Author's name: Perignon M., Sinfort C., El Ati J., Traissac N., Drogue S., Darmon N., Amiot M.-J. and the Medina Study Group Title: How to meet nutritional recommendations and reduce diet environmental impact in the Mediterranean region? An optimization study to identify more sustainable diets in Tunisia. Journal: Global Food Security 23:227–235 https://doi.org/10.1016/j.gfs.2019.07.006

**Table:** 

**Value of the Data** • Our paper provides data describing the worldwide environmental impacts of water consumption and land use for food items consumed in France and Tunisia. Indeed, although many data are available for potential impacts on climate change, very few data exist for water consumption and land use impacts. Beyond the data itself, we propose a calculation method and data sources that can be transposed to other countries in the world. • The present data will benefit to (i) all those interested directly by the data of foods to evaluate the environmental impacts of diets in Mediterranean countries but also in other ones, (ii) those seeking case studies of the impact assessment methods that have been used here and (iii) more generally, these data can be useful for research works in accordance with food security and climate change within the frame of the 2030 Agenda Sustainable Development (SDG). • Data can be directly used to compute dietary food impacts with various scenarios. Intermediary data and the computation method can be used to compute equivalent data in other countries. • Our publication proposal includes feedback and a critical analysis of the method and data that will be valuable for scientists who would like to carry out an equivalent approach.

## 1 Data

The data provided describe water consumption, Land Use and associated impacts per country for 1 kg of food item used in France and Tunisia. The origin of food item is considered to compute impacts on all countries of the world. The data are used in Perignon et al., 2019 [1].

## 2 Data description

The dataset is an Excel file with two main worksheets:

i. Data per kg of item and per country of origin for France
ii. Data per kg of item and per country of origin for Tunisia

These worksheets give the amounts of used resources (water and land use surface) and potential environmental impacts for 1 kg of each product in all the countries of origin for the food items available in France and in Tunisia.

Supplementary information provide intermediate data used for the computation: list of food items with their coding in other databases, codes for imported food items, list of animal feed items with their coding in other databases, list of countries with classification, land use coding.

## 3 Data analysis

**Fig. 1:**
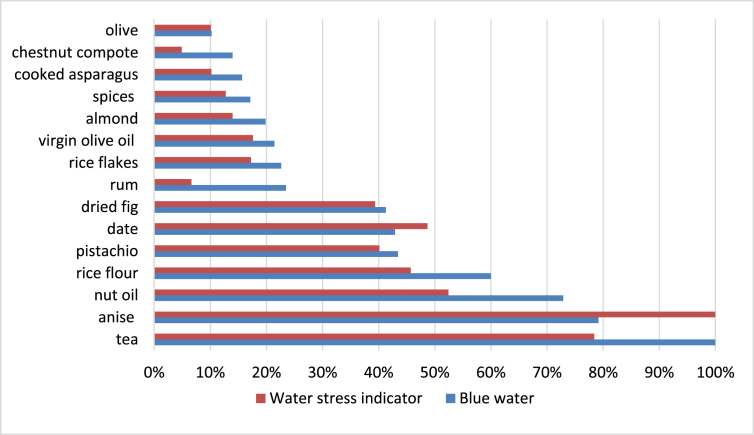
Normalised values of blue water consumption and relative water stress for food items most concerned. Case of France.

**Fig. 2:**
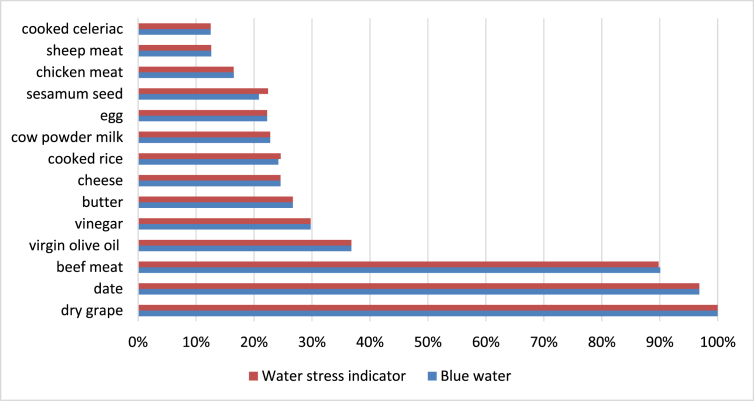
Normalised values of blue water consumption and relative water stress for food items most concerned. Case of Tunisia.

**Fig. 3:**
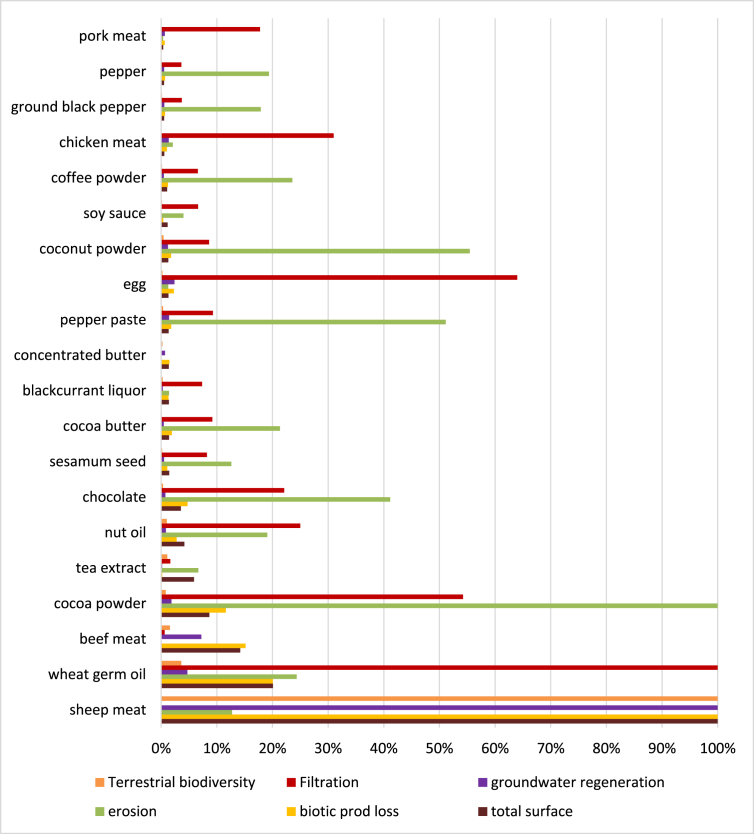
Normalised values of land use surfaces and relative impacts for food items most concerned. Case of France.

**Fig. 4:**
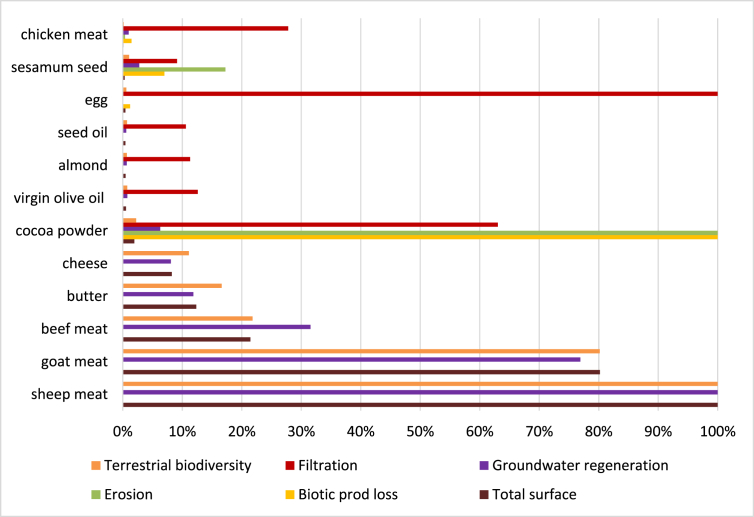
Normalised values of land use surfaces and relative impacts for food items most concerned. Case of Tunisia.

Food ingredients having the most important values for consumption (water or land use) and for their relative potential impacts were reported Fig. 1, Fig. 2, Fig. 3, Fig. 4 for France and Tunisia. Values were normalised with the maximum value of each category in order to allow comparisons: a 100% value means that the corresponding food ingredient have the maximum score, compared with the other food ingredients. Indeed, values by themselves do not have a lot meaning.

Only food ingredients with at least one value greater than 10% have been reported in these charts. Food items are sorted with consumption values (water or land use).

Differences in water consumed and water deprivation stress arises from the origin country of the ingredient. For instance, for French consumed products, tea is the ingredient that uses the highest amount of blue water per kg. Nevertheless, the product that generates most water stress per kg is anise. As most ingredients consumed in Tunisia are produced in this same country, the values of both indicators are rather similar. For products consumed in France, high values are obtained for tea, seeds (anise, spices), nuts (nut oil, pistachio, almond, chestnut compote), rice derivatives (rice flour, rice flakes), dried fruits (date, dried fig), rum, olives (and olive oil) and asparagus. Same trends are observed for products consumed in Tunisia but animal products (meat, cheese, milk, eggs) are also important, mainly because of feed that is produced locally.

For land use and relative potential impacts, in both countries, high surfaces are required for animals bred in pasture (goat, sheep, beef). In most countries of origin, effect on filtration is relatively higher when the surfaces are reduced (e.g. egg, pork meat). For tropical products, effect on erosion is usually high.

## 4 Data quality

### 4.1 Completeness

Food products were identified from nationally representative individual food consumption surveys conducted in France [2,3] and Tunisia [4]. These surveys captured the main items to be considered; however, some items that are consumed less frequently may not have been integrated. Source data were found in the FAOstat dataset [5], UNComtrade statistics [6], Water footprint datasets [7,8] and characterization factors for water consumption from Pfister [9] and for land use impacts from the LANCA method [10,11] and from Chaudhary [12].

Some data were lacking in the FAOstat yield dataset. When the origin ratio was higher than 1%, we identified proxies. This concerned two products: root tubers (replaced by carrots, when missing) and pepper (sometimes referenced in green, and sometimes in dry form). In the other cases, we set the yield to 0. We paid special attention to sugar because it can be produced from beetroots or sugarcane. The default value was beetroot and, when we could not find the relevant yield, we replaced it with the sugarcane yield.

The water footprint database contains empty cells that could be missing values or 0 values. As we could not differentiate between these two options, we replaced empty cells with a 0 value.

We did not identify any missing data in the CF tables (WSI, LANCA or biodiversity CF's).

### 4.2 Source data reliability

For food production, the FAOstat database provided indications about the data reliability. In the vast majority of cases the collected data were categorized by FAO as “official data”. Data on trade were from UN Comtrade, the United Nations Statistical Division database. UN Comtrade data come from official international trade statistics, and various sources of misreport may affect their reliability. As we only focused on imports, the sources of errors were multiplied by the number of declarants, as discussed in Drogué and Bartova [13]. In both databases, we identified and fixed some input errors (e.g., missing digits). Other similar errors may not have been removed.

For the other datasets used (Water Footprint, LANCA, WSI), no indication was given about data quality.

### 4.3 Accuracy

The amount of consumed resources and the potential impacts were computed for the average country annual values or for more aggregated values. For instance, biodiversity CFs considered only three types of agricultural land use (annual, perennial, and pasture). The estimation of indirect land use by animal breeding also was very rough. For agricultural production, the values could vary significantly depending on the month of the year [14] and on the climatic region. Applying LANCA CFs at the country level is a rough approach, usually reserved for background processes. However, currently, it is impossible to know exactly the production basins in each country for each product, although data for resource consumption are available at this level. We tried to describe the production conditions as accurately as possible, considering terrestrial biomes for instance. Nevertheless, the values provided here should be considered as proxies, and may include important biases.

### 4.4 Representativeness

As previously underlined, UN Comtrade provides quantities recorded by customs officers, and we only considered the import values. With the exception of some tropical products, we considered only the last trade movement. To compute the provenance ratio of each product, we collected data on import and production. In these ratios, we considered French and Tunisian productions without subtracting the exports, and therefore, in the dataset, the national production was generally overestimated.

Setting the land use surface to 0 for water and for seafood products is a questionable choice. Indeed, seafood consumption has critical effects on biodiversity that were not taken into account in our dataset (only terrestrial biodiversity was considered here).

Finally, we did not take into account the losses occurring between the production field and the consumer purchase, although they can be rather high (FAO estimated that about one third of the food produced in the world is lost). From this point of view, all impact data provided in our dataset are underestimated.

### 4.5 Consistency

Some inconsistency could have arisen from the fact that the data used to generate the datasets were collected at different times. Indeed, the Tahina project gathered data from 2005 to 2009, the INCA survey described food consumption in 2006 and 2007, and FAOstat and UN Comtrade data were for 2011. The water footprint data were published in 2010 and 2011, the WSI CFs in 2009, and LANCA data in 2016. For UN Comtrade data, we verified that the date discrepancy did not significantly affect the results.

Consistency between databases was an important challenge. As each database used its own codes and names, we had to match the different codes of a given product (food or ingredient). The difficulty lied on the different classifications related to the different objectives of each database (food consumption, commercial or crop groups). Consequently, we had to specifically identify each product in each dataset. The correspondence was not always bijective. For instance, consistency with the UN Comtrade dataset was made possible by identifying all the commodities corresponding to each food product (see Material and Methods). The selection of these commodities may have not been exhaustive and our list can certainly be improved. Other difficulties appeared when a food item had more than one raw product: this is the case of sugar, as explained above.

## 5 Experimental design, materials, and methods

### 5.1 Identifying food items

We extracted the food products consumed in France from the Second French Individual and National Study on Food Consumption INCA2 [2,3], and in Tunisia from the National Food Consumption Survey carried out in the framework of the Tahina project (Epidemiological Transition And Health Impact In North Africa [4]). We broke down processed food products into ingredients based on recipes provided in the respective food consumption surveys. We included all ingredients and raw food products in a list of 842 items. By grouping analogous items, we obtained a list of 347 items (Supplemental Information “2.Food_Items”) among which 96 items were used in both countries, 244 were specific to France, and 7 to Tunisia.

### 5.2 Identifying the origin of each food item

For each food item and for both countries (France and Tunisia), we extracted the amount of the corresponding commodity imported per year from the UN Comtrade database [6]. Each item was related to one or more commodities referenced using Harmonized System (HS) codes (e.g., milk can be imported as liquid milk or milk powder; meat with or without bones). The internationally standardized HS coding system includes names and numbers to classify traded products, defined by the World Customs Organization. The selected reference year was 2011. When a food item was related to several HS codes (several forms), we considered all of them, and selected one as the reference imported commodity. Then, we defined the mass product ratio (r_ref) between the reference commodity and the imported commodity as follows:

r_ref=(imported commodity [kg])/(reference imported commodity [kg])

For instance, the food item “potato flakes” can be imported as “Potatoes other than seed potatoes, fresh/chilled” (HS code: 070190) or “Flakes, granules & pellets of potatoes” (HS code: 110520). The selected reference form was “Potatoes other than seed potatoes, fresh/chilled”, and the r_ref was 1 for “Potatoes other than seed potatoes, fresh/chilled” and 0.28 for “Flakes, granules & pellets of potatoes”. The imported commodities corresponding to each food item, the reference imported commodity, and the r_ref values are provided in Supplemental Information “3. Imported_items”.

We obtained the amounts of commodities produced in France and Tunisia from the FAOstat database [5]. Then, all the amounts were converted in equivalent reference commodity for each country, and finally the percentages of origin in mass per country (origin ratio) were obtained. It should be noted that FAO categories are less detailed than the UN Comtrade categories. Thus, when considering the amount of production of a given food item, several UN Comtrade commodities could be included. Consequently, the production amounts of France and Tunisia were overestimated. We did not integrate the amounts exported from France and Tunisia.

Some items, mainly tropical products, are imported through intermediary countries. For instance, most rice imported in France comes from Italy (1st), Spain (2nd), Netherlands (3rd) and Belgium (4th). For these items, we extracted the actual production origin (e.g., India, Pakistan, Thailand, Cambodia for the rice imported from Italy) from the UN Comtrade database, and computed the origin ratio with the correct values.

### 5.3 Product losses

We did not take into account the losses along the production chain. We computed all data for 1 kg of food item, including the non-edible parts (fruit stones, peals, etc.), and before cooking (for instance pasta, rice or lentils were considered dried products).

### 5.4 Animal items

For animal items, we took into account the resources consumed directly by the animals and those required for feed production. Cattle and swine breeding in France and ovine breeding in Tunisia are mainly for consumption within the country (see FranceAgriMer, 2012 [15], for France). Given the dietary habits of both countries, we assumed that animal feed items were consumed by animals produced mainly in France and Tunisia, respectively. For France, the main data on animal feed came from a national study [16] with complementary data for poultry production [17]. We considered only supplemental intakes (not grazing that was counted in the directly consumed resources). We obtained Tunisian animal feed data from Tunisian experts [18]. The list of feed items considered for our dataset is in Supplemental Information “4.Animal_Feed” for the main animal types. Then, we evaluated the origin of feed products as described in 1.2.

### 5.5 Water consumption, land use, and the associated impacts

#### 5.5.1 Water consumption

In LCA-based studies, water consumption is seen as the amount of water that does not return to the local water cycle, and it is defined as the amount of water evaporated during the production phase or included within the crop or animal products. Several methods have been proposed in the literature to estimate these quantities. We chose the Blue Water values obtained from the Water Footprint database for crop [7] and animal products [8] for each country. The Blue Water data describe the water brought to the plants in conditions of no water stress (the values to fulfill the plant needs were obtained with the FAO Cropwat model), and may overestimate the amount of water actually consumed. In this database, products are also referenced with HS codes. When we could not find the HS code of an item in the Water Footprint database, we identified an equivalent product, and then calculated the mass ratio between these products (r_prod_bw in Supplemental Information 2. Food_Items). For instance, lime pulp food item (HS code 40) was linked to citrus fruits in the Water Footprint dataset with a mass ratio of 0.5.

In the Water Footprint database, data are given for each product and the corresponding raw product, when appropriate. In this case, both are linked with a “product fraction”. When available, we used these product fraction values to calculate the r_ref (see chapter 1.2). For animal products, the Water Footprint database integrates both direct (animals) and indirect (feed) water needs.

Fish and seafood products are not included in the Water Footprint database because the corresponding water consumption is negligible, but a value of 0 can induce errors during further data processing. By visiting a Mediterranean harbour (Sète, France), we evaluated the amount of lost water per kg of seafood (Table 1). We estimated that the freshwater loss (returned to the sea) did not exceed 5 L per kg of seafood; this included the water used in fishing activities and fish processing (Table 1). It is a very low value compared with that of all the other food items. Therefore, we considered that this value was a good proxy that did not need to be improved.

**Table 1:** Water consumption for fish production.

Amount of lost freshwater
**Fishing activities 1 to 2 L/kg**
Ship washing ∼1
Ice production 0.2
**Fish trade activities 1 to 2 L/kg**
Cutting plant 0.7
Brine production 0.3
Ice production 0.2
**Total estimated water consumption <5 L/kg**

#### 5.5.2 Occupied land surfaces

We computed the surface needed to produce 1 kg of item from the yield value in kg/ha. For crop productions, we extracted the yield values in each country of origin from the FAOstat database [5] for 2011. We gave a surface value of 0 when the product-country pair was absent, and also for seafood and water.

For animal products, land use can be direct (grassland) or indirect (surface needed for feed production). For indirect land use, we computed the land surface use with the method implemented for crop production. For ruminant direct land use, we adapted the methodology proposed by Ref. [8]. It is based on the study by Bouwman [19] that gave the production (kg) per hectare of grassland of beef, milk and mutton and goat meat for pastoral (P) and mixed + landless (M + L) production systems for different world regions. The repartition of countries in these regions is given in Supplemental Information “5.Country”. We used the off-take values also provided in this study to derive the percentage of production from P and M + L systems. We made the strong hypothesis that milk production is shared between P and M + L systems with the same ratio as for cattle production. For pigs and poultry, we used the mean values of 1 pig/m^2^ and 22 fowls/m^2^, and the carcass yield values provided by FAOstat.

### 5.6 Impact indicators

In LCA, potential impacts are calculated by multiplying the amount of consumed water and occupied land surfaces by impact factors called characterization factors (CF). For water deprivation and land use, the pathways between resource consumption and potential impacts are still under discussion in the scientific community, and several methods are considered. We selected methods that already provide CF for all countries and are recognized by scientists.

#### 5.6.1 Water deprivation potential impacts

The selected CF was the Water Stress Indicator (WSI) provided by Pfister, Koehler, and Hellweg [9] for each country in the world. This indicator is based on a modified withdrawal-to-availability (WTA) ratio to provide values between 0.01 and 1 of deprived water m3/consumed water m3 (1 for the maximum stress). This indicator was computed at the grid cell level (0.5° × 0.5°) and is available for every month of the year. Here, we used averaged values per year and per country. In their review about impacts on resources, water and land Life Cycle Impact Assessment (LCIA) methods, Sala et al. [20] compared the WSI method with other methods and found that “the model performs well in terms of applicability and robustness”. They underlined that the WTA approach is more representative for human impacts (rather than ecosystems). Other limits are also listed. International experts of the UNEP/Society for Environmental Toxicology and Chemistry WULCA working group (http://www.wulca-waterlca.org/) published a more recent method (Available Water Remaining, AWARE); however, it is less straightforward because it provides “a surface-time equivalent to generate unused water in a given region” (m2.month.m-3) [21].

#### 5.6.2 Land use impacts

Land use impacts are due to soil transformation and soil occupation. Our scope was to evaluate the main threats due to soil occupation in all the countries impacted by food production for consumption in France and Tunisia. For soil occupation, among the approaches compared by Vidal-Legaz et al. [22], we selected the LANCA method [10,11] that is recommended by the Joint Research Center (the European Commission's science and knowledge service) for the assessment of the environmental footprint of land use in LCI [23]. The method provides characterization factors at country level for the following processes: erosion, infiltration reduction, physicochemical filtration reduction, groundwater regeneration reduction and biotic loss production. For soil transformation, we computed the potential impacts on terrestrial biodiversity using the method described by Chaudhary et al. [12]. The global CFs from this study have been provisionally recommended by the UNEP-SETAC Life Cycle Initiative organization.

a) LANCA (soil occupation)

In addition to describing the method, the LANCA report provides averaged CFs for 58 land use types, including 24 agricultural types for land use transformation and occupation, for all countries worldwide. We only selected CFs concerning soil occupation. To determine the land use type for each food component, we gathered food items into 23 product groups (Supplemental Information “2.Food_Items”, in the column ‘crop_group’), and countries into the 14 terrestrial ecoregions defined by the World Wildlife Fund (https://www.worldwildlife.org/biomes). Country repartition in biomes is given in Supplemental Information “5.Country”. Then, we associated each product group-ecoregion combination to one of the LANCA land use types (see Supplemental Information “6.LandUse_code”). The biome 0 code was given to non-existing associations. CFs are given for five potential impacts: erosion potential (EP), infiltration reduction potential (IR), physico-chemical filtration potential (PF), groundwater regeneration reduction potential (GR), and biotic production loss potential (BP). As Sala et al. (2016) demonstrated that the IR and PF values are highly correlated (Pearson's correlation factor = 1), the IR factor was not provided in our dataset.

b) Biodiversity

The Chaudhary method uses the countryside species-area relationship (SAR) to quantify regional species loss for five taxa and six land use types in 804 terrestrial ecoregions. We used the aggregated CFs per country, including all taxa. Among the six land use types, we distributed food item productions within three agricultural types: annual crops, permanent crops and pasture (biodiv_group in Supplemental Information “2.Food_Items”).

### 6 Data analysis

Total amounts of consumed water and total surfaces of land use were compiled for 1 kg of each ingredient consumed in France and in Tunisia. Total relative impacts were also computed. Each of these values were normalised by the maximum value among all the ingredients.

Mean values per product group were not provided because groups were built with a food point of view that does not meet agronomic considerations. Due to this, standard deviations in each category are really important and average values does not have any sense.
